# Heat shock pathway activation by TRC051384 protects spiral ganglion neurons from noise-induced hearing loss

**DOI:** 10.1172/jci.insight.203285

**Published:** 2026-08-25

**Authors:** Jintao Yu, Miguel A. Ramirez, Yi-Zhi Wang, Seby Edassery, Maxwell Shramuk, SangEun Yeom, Casey Jiaxi Li, Yuvraj Joshi, Mary Ann Cheatham, Mark A. Rutherford, Leah J. Welty, Jeffrey N. Savas

**Affiliations:** 1 Department of Neurology,; 2 Department of Psychiatry and Behavioral Sciences, and; 3 Department of Preventive Medicine, Northwestern University Feinberg School of Medicine, Chicago, Illinois, USA.; 4 Department of Communication Sciences and Disorders and; 5 The Hugh Knowles Center for Clinical and Basic Science in Hearing and Its Disorders, Northwestern University, Evanston, Illinois, USA.; 6 Department of Otolaryngology, Washington University in St. Louis School of Medicine, St. Louis, Missouri, USA.

**Keywords:** Aging, Neuroscience, Otology, Chaperones, Synapses, Transcriptomics

## Abstract

Noise-induced hearing loss (NIHL) is a major public health problem caused by damage to cochlear hair cells, synapses, and spiral ganglion neurons (SGNs). Since effective treatments are lacking, we investigated cellular stress responses induced by moderate and loud noise in a mouse model of cochlear synaptopathy. RNA-seq and spatial transcriptomics revealed that noise exposure elicited a robust but transient upregulation of endoplasmic reticulum chaperones and proteasome subunits in SGNs and their supporting cells. To target this response, we administered TRC051384, a small-molecule activator of the heat shock transcription factor Hsf1, prior to noise exposure. TRC051384 crossed the blood-labyrinth barrier and reached the cochlea, induced heat shock protein gene expression, and restored ubiquitin-proteasome function in SGNs. Notably, TRC051384 treatment enhanced auditory brainstem response threshold recovery, preserved Wave I amplitudes, and maintained ribbon synapse density. Together with the existing literature, these findings identify proteotoxic stress in SGNs as a contributor to noise-induced hearing loss and support pharmacological activation of HSF1 as a promising therapeutic strategy.

## Introduction

Noise-induced hearing loss (NIHL) is a prevalent condition affecting greater than 5% of the global population, yet no effective therapies are currently available (1, 2). Exposure to harmful levels of noise is almost unavoidable in the industrialized world (3–5). NIHL is the result of a complex interplay of variables, including exposure duration, sound intensity, and frequency, in addition to individual genetic and physiological differences (2). NIHL can result from exposure to an acute intense acoustic event, which causes immediate and irreversible damage to the inner ear. In contrast, chronic exposure to moderately loud sounds (typically above 85 dB SPL but below 100 dB SPL) often leads to subtle, progressive auditory deficits. Although these deficits may not be detected by standard audiometry, they are increasingly recognized as common and clinically significant and substantially increase the prevalence of NIHL (6, 7).

Loud noise adversely affects multiple cochlear structures, cell types, and cellular processes. For example, intense auditory stimulation imposes a high metabolic demand on the cochlea (8), leading to increased oxygen consumption, excessive free radical production, and cellular stress — all of which can contribute to cell death (9–11). In addition, noise exposure has been shown to reduce both tectorial membrane calcium levels and cochlear blood flow (10, 12), further exacerbating cochlear dysfunction. Hair cell (HC) death can occur through a variety of signaling pathways triggered by these stressors (13). Moreover, loud noise can lead to metabolic decompensation, characterized by nuclear and mitochondrial swelling, cytoplasmic vesiculation (14), and ultimately apoptosis (15). Noise exposure can result in either permanent threshold shifts (PTS) due to irreversible damage, or temporary threshold shifts (TTS) that recover within a few weeks. The primary targets of noise-induced damage in the cochlea are supporting cells, cells of the lateral wall, the inner and outer HCs (IHCs, OHCs) and their stereocilia bundles, and IHC ribbon synapses onto auditory nerve fiber (ANF) terminals (16, 17). ANF terminals can physically detach from the presynaptic ribbons synapses of IHCs following noise exposure, but partial reattachment and functional recovery are sometimes observed (18–21). However, loud noise exposure can lead to the permanent loss of up to 50% of IHC ribbon synapses — even in cases where standard audiograms appear normal, which is a condition referred to as cochlear synaptopathy (22–25).

In our previous studies, we used quantitative, tandem mass spectrometry–based (MS-based) proteomic analysis to study cochlear extracts from mice immediately after they were exposed to loud noise. We discovered that noise levels sufficient to induce hearing loss caused an extensive protein accumulation in the cochlea (26). The level of the full proteasome complex, along with a broad spectrum of protein chaperones, was elevated. However, we mapped only a handful of elevated proteins to specific cochlear cell types. Furthermore, the interpretation of these results was complicated by the fact that the sound pressure level (SPL) of the noise exposures caused compound threshold shifts, including both a temporary and a permanent component. This makes it difficult to distinguish adaptive cellular responses from ineffective compensation for structural damage. In additional MS-based proteomic analyses, we found that the translational machinery (i.e., ribosomal protein sub-units) was selectively elevated during at 2 weeks after noise exposure, thereby suggesting that loud noise (94 dB SPL, 0.5 hour, 8–16 kHz) triggered a burst of protein turnover that may play a key role in restoring hearing sensitivity (27).

Building on these findings, the present study primarily investigated a mouse model of cochlear synaptopathy. After validating the noise exposure paradigm, we performed discovery-based bulk RNA-seq to characterize the timeline of gene expression induced by noise exposure causing cochlear synaptopathy. Notably, a coordinated peak in gene expression was observed 4 hours after the 2-hour exposure to 100 dB SPL. Pathway enrichment analysis of the significantly elevated genes revealed a striking enrichment of the endoplasmic reticulum (ER) chaperone complex, emerging as the most significantly overrepresented term by a wide margin. This was driven by the increased gene expression of several protein chaperones, including *Hsp90b1* and *Hspa5* encoding Endoplasmin and BiP, respectively. Spatial transcriptomics revealed that both spiral ganglion neurons (SGNs) and their associated glia cells exhibited elevated expression of genes encoding proteasome subunits. Next, we examined whether preactivating the heat shock response, a pathway activated following stressors that cause protein misfolding, could protect against noise-induced damage. To do so, we pharmacologically activated the transcription factor Hsf1 prior to noise exposure, using the previously identified small molecule TRC051384 (28). Using a ubiquitin-proteasome system (UPS) activity reporter mouse line, we observed that cellular stress in response to synaptopathic noise exposure was primarily localized to the spiral ganglion. Notably, pretreatment with TRC051384 mitigated the noise-induced accumulation of a GFP-based reporter, indicating enhanced proteostasis and improved protein degradation capacity following noise-induced stress with activation of the heat shock response. Finally, we found that the prophylactic administration of TRC051384 not only improved the recovery of auditory brainstem responses (ABR) Wave I amplitudes but also conserved synaptic density.

## Results

### Moderate noise exposure causes cochlear synaptopathy in 16-week-old CBA/CaJ mice.

ABRs are sound-evoked potentials that can be used to determine abnormalities or deviations in hearing sensitivity. The first wave in the ABR represents the synchronous auditory nerve discharge at the onset of sound, proportionate to activity of the cochlear ribbon synapses between IHCs and SGNs. Initial experiments aimed to replicate the previously reported “hidden hearing loss” phenotype in CBA/CaJ mice (22). Thus, we exposed CBA/CaJ mice at 8 and 16 weeks of age to 8–16 kHz octave-band noise at 100 dB SPL for 2 hours. ABRs and distortion product otoacoustic emissions (DPOAEs) were measured at baseline and at 1, 3, and 14 days after noise exposure. Both the 8- and 16-week-old mice exhibited significantly elevated ABR thresholds (i.e., the lowest tone level necessary to generate the ABR waveform) after noise. The largest threshold shift, 40 dB, was observed for tones of 32 kHz. Elevated thresholds persisted at frequencies above 20 kHz for at least 3 days after noise (DAN). ABR thresholds recovered to near baseline levels by 14 DAN in 16-week-old but not in the younger 8-week-old CBA/CaJ mice (Figure 1A and Supplemental Figure 1A; supplemental material available online with this article; https://doi.org/10.1172/jci.insight.203285DS1). In 16-week-old-mice, Wave I amplitudes in response to 8, 16, 24, and 32 kHz tones remained significantly reduced (~50% at frequencies > 16 kHz) at 14 DAN (Figure 1, B and C, and Supplemental Figure 1, B–D). This lasting reduction in ABR Wave I amplitude following a TTS is consistent with permanent cochlear synaptopathy. Notably, we found that, for the 8-week-old mice, Wave I amplitudes failed to recover to prenoise levels at frequencies greater than 16 kHz (Supplemental Figure 1, E–G).

DPOAEs are acoustic responses generated by vibrations along the basilar membrane when 2 pure tones stimulate the cochlea. These emissions reflect activity of the cochlear OHCs. Elevation of DPOAE thresholds indicates dysfunction of the OHCs, upstream of auditory nerve excitation by ribbon synapses. DPOAE thresholds were elevated at 1 DAN in both ages of mice (Supplemental Figure 1, H–N). In contrast to ABRs, DPOAE thresholds and amplitudes fully recovered at all tested frequencies by 14 DANs, indicating that OHCs were not permanently damaged by these noise exposures in either group of mice.

In the absence of threshold shifts, a reduction of ABR Wave I amplitude indicates damage or loss of cochlear ribbon synapses. To verify this finding, we examined immunostained whole mounts of the organ of Corti with antibodies against CtBP2 and GluA2, which stain presynaptic ribbons and postsynaptic receptors, respectively (Figure 1D). Juxtaposition of these 2 markers indicates a synapse. Consistent with our ABR data at 14 DAN, we found significant reductions in cochlear ribbon synapse density at all frequencies above 20 kHz (Figure 1E). In the 32 kHz region, the average number of ribbon synapses per IHC was 15.0 ± 1.1 in cochlea from age matched unexposed CBA/CaJ mice and 6.0 ± 1.1 at 14 DAN. Similar results were obtained when separately counting either presynaptic ribbons or postsynaptic puncta (Supplemental Figure 1, O and P). Together, our results confirm that this level of noise exposure produced a TTS while inducing cochlear synaptopathy that is thought to be permanent in CBA/CaJ mice.

### A wave of protein chaperone gene expression peaking 4 hours after synaptopathic noise.

We next sought to identify cellular pathways engaged acutely after synaptopathic noise exposure. We repeated our noise exposure paradigm in a new cohort of 16-week-old mice and harvested total cochlear RNA at 0, 4, or 8 hours after noise. Bulk cochlear gene expression was measured using paired-end RNA-seq analysis (29). We obtained greater than 40 million mapped reads at a total mapping rate of > 96.91% from each of the 3 biological replicates per group. To investigate the reproducibility of our transcriptomic analysis, we assessed the correlation of gene expression patterns between the individual biological datasets using t-distributed stochastic neighbor embedding (t-SNE). Notably, the biological replicates clustered according to the duration after exposure (Figure 1F).

We assessed changes in gene expression at each time point relative to unexposed controls, identifying 158, 118, and 74 genes with significantly elevated levels at 0, 4, and 8 hours after noise, respectively (Supplemental Table 1). To identify the pathways engaged in response to this noise exposure paradigm, we performed Gene Ontology (GO) cellular component (CC) enrichment analysis using DAVID (30). Among all 3 replicates, the most significantly enriched term was the GO:CC category endoplasmic reticulum chaperone complex (GO:0034663), which was detected only at 4 hours after noise (Figure 1G and Supplemental Table 2). This 159.7-fold enrichment of the ER chaperone complex was 7-fold greater than the second most enriched GO:CC term. To visualize noise-induced changes to cochlear gene expression, we graphed the fold changes observed 4 hours after noise exposure relative to unexposed controls on a volcano plot (Figure 1H). The 5 genes in our dataset associated with GO:0034663 were *Hsp90b1*, *Hspa5, Dnajb11*, *Dnajc13*, and *Sdf2l1*. Close inspection of gene expression time-course revealed a slight elevation immediately after noise, peaking 4 hours after noise, and resolving to near baseline levels by 8 hours (Figure 1I). The protein products of these genes play well-known and important roles in regulating protein quality control (QC). *Hsp90b1* (i.e., endoplasmin) is an ER-resident chaperone, functioning in protein folding and QC. *Hspa5* (i.e., BiP) is the master chaperone in the ER and is central to the unfolded protein response (UPR). *Dnajb11* (i.e., ERdj3) is an ER cochaperone that works with BiP to assist in protein folding. *Dnajc13* (i.e., RME-8) a cochaperone involved in endosomal trafficking and implicated in membrane dynamics. *Sdf2l1* encodes an ER stress–induced protein, also participating in protein folding. These ER chaperones induced by noise are canonical targets of the UPR. We therefore asked whether noise also engaged, Hsf1-dependent arm of the proteostasis network. The level of Hsf1 mRNA increased approximately 1.8-fold after noise exposure relative to unexposed controls (*P* = 0.026), with a comparable increase immediately after noise and at 4 and 8 hours. However, it’s worth noting that Hsf1 is primarily regulated posttranslationally. Hspa1a, a cytosolic HSF1 target, did not differ significantly from controls at any time point (*P* > 0.4; Figure 1J).

To investigate whether expression of the ER chaperone genes is upregulated in all cells or in a select group of cells following exposure, we utilized RNAscope based in situ hybridization to visualize patterns of *Hsp90b1*, *Hspa5*, and *Sdf2l1* gene expression after noise exposure. Expression of all 3 genes was markedly elevated in the spiral ganglion, IHCs, and OHCs at 4 hours after noise (Supplemental Figure 2). Interestingly, *Hsp90b1* expression also appeared to increase around the spiral limbus. Thus, RNAscope in situ hybridization revealed that the primary sensorineural cells contributed to the elevated expression of genes involved in ER protein QC in the cochlea.

### GeoMx spatial transcriptomics reveals elevated proteasome subunit gene expression in SGNs and support cells following noise exposure.

To further investigate the transcriptional response of cochlear cells to noise exposure, we performed GeoMx spatial transcriptomic profiling on cochlear sections from mice exposed to 100 dB SPL for 2 hours, and the cochleae were harvested 4 hours after exposure, alongside mock-exposed controls. Immunofluorescence imaging identified NF200^+^ SGNs, which were segmented into regions of interest (ROI) for GeoMx spatial transcriptomic analysis. Each ROI was further subdivided into a neuronal mask (i.e., NF200^+^) and a supporting cell mask (i.e., NF200^–^) to distinguish expression profiles between SGNs and ganglionic supporting cells (Figure 2, A–F, and Supplemental Table 3). Dimensionality reduction using t-SNE revealed that RNA expression profiles clustered primarily by cell type and, to a lesser extent, by noise exposure condition (Figure 2G). In SGNs, noise exposure induced substantial upregulation of genes encoding the proteostasis network, particularly multiple subunits of the proteasome (Figure 2H). Consistently, GO enrichment analysis of the upregulated genes identified the proteasome complex as the only CC category significantly enriched (Figure 2I and Supplemental Table 4). Similarly, support cells also exhibited noise-induced upregulation of mRNAs encoding proteostasis factors, with GO enrichment analysis identifying significant enrichment in the proteasome complex and nucleus (Figure 2, J and K, and Supplemental Table 4). Taken together, these data suggest that both SGNs and their supporting cells activate proteasome-associated transcriptional programs in response to synaptopathic noise.

### TRC051384 induces heat shock response.

The heat shock response and the ER UPR are 2 parallel arms of the proteostasis network that are frequently coactivated by a single stressor and show documented bidirectional crosstalk (31, 32). Having found a robust induction of ER chaperone transcripts, we therefore examined the possibility of engaging the cytosolic Hsf1-dependent arm. TRC051384 is a small-molecule activator that robustly induces the cellular heat shock response. It belongs to the 2-propen-1-one chemical class and functions as a potent inducer of heat shock protein (Hsp) expression via activation of Hsf1 (28). To verify that TRC051384 effectively engages canonical Hsf1 signaling, we examined the transcriptional activation of *Hspa1a*, a well-characterized Hsf1 target gene. HEK293T cells were treated with either vehicle control or TRC051384, and transcript levels were quantified by qPCR. TRC051384 treatment led to a dramatic (>20-fold) increase in *Hsp1a* expression at the 4-hour time point compared with vehicle-treated cells, while *Hsf1* mRNA levels remained unchanged, consistent with the mechanism of posttranslational activation of Hsf1 (Figure 3A). To determine whether this transcriptional response could be reproduced in inner ear cells, we performed qPCR on cochlear organotypic cultures derived from P4 rat pups. Remarkably, a similar expression pattern was observed, with *Hspa1a* showing its strongest induction at the 4-hour mark (Figure 3B), albeit with a smaller fold change than in HEK293T cells. This attenuated response in cochlear tissue may reflect cell type–specific differences in basal Hsf1 activity, accessibility of the *Hspa1a* promoter, or pharmacokinetic factors influencing compound uptake. Together, these findings confirm that TRC051384 is a potent pharmacological activator of the heat shock response in both immortalized human cells and ex vivo mammalian cochlear tissue, reinforcing its utility as a molecular tool for inducing Hsf1-mediated stress protection pathways.

To evaluate the pharmacokinetic accessibility of TRC051384 to the inner ear, we examined its ability to penetrate the cochlea after systemic administration. Mice received i.p. injections of TRC051384 (60 mg/kg). Then, the cochleae were dissected and processed to solubilize small molecules. We analyzed TRC051384 alone and in the resulting cochlear extracts using MS1 targeted selected ion monitoring (tSIM) (33), to sensitively and selectively quantify TRC051384 based on its molecular mass (465.23 Da; +2 charge state = *m/z* 233.6261). Evidence of drug penetration was clearly observed; extracts from the cochleae of TRC051384-treated mice displayed a strong peak at *m/z* 233.62, which eluted at 30–31 minutes. This signal was entirely absent from the extracts of vehicle-treated controls (Supplemental Figure 3, A and B). To further confirm the identity of the compound within the complex biochemical environment of the cochlea, we performed a fragment ion search by coupling tSIM with data-dependent MS2 acquisition. This analysis revealed numerous diagnostic TRC051384 fragment ions, unambiguously verifying its presence in cochlear tissue (Supplemental Figure 3, C and D). These findings demonstrate that TRC051384 readily crosses the blood-labyrinth barrier and accumulates in the cochlea at quantifiable levels, establishing its pharmacological accessibility to inner ear tissues. This property provides a strong foundation for exploring TRC051384’s ability to target heat shock pathways as a potential therapeutic strategy for auditory protection and repair.

### TRC051384 alleviates noise-induced impairment of the ubiquitin proteasome system (UPS) in SGNs.

Our prior work showed that acoustic exposures at 94 or 105 dB SPL elicit pronounced increases in cochlear protein ubiquitylation accompanied by elevated levels of proteasome subunits (26). To follow up on this finding, we acquired the UPS activity reporter mouse line, Tg(CAG-Ub*G76V/GFP)1Dant, which broadly expresses the green fluorescent protein (GFP) fused to a constitutively active degradation signal (i.e., UbG76V) (34). Under normal circumstances, the GFP protein is readily ubiquitinated, marking it for degradation soon after the protein is synthesized (Figure 4A). However, GFP accumulates and fluorescence increases when the proteasome is impaired or overwhelmed. Thus, to determine which cochlear cells have impaired UPS in response to 94 or 105 dB SPL noise, we examined GFP fluorescence in cochlear midmodiolar sections. The 105 dB SPL intensity is included to investigate if intense noise causing PTS causes an even robust accumulation of GFP compared with 94 dB SPL. We discovered that the GFP selectively accumulated in SGNs in a noise intensity–dependent manner (Figure 4, B and C). One potential caveat to this interpretation is that the expression of the *GFP* gene may vary among different cochlear cell types. To rule out this possibility, we examined the distribution of the *GFP* mRNA within the cochlea using RNAscope in situ hybridization. Notably, we detected *GFP* mRNA expression among various cell types within the spiral ganglion, the organ of Corti, the spiral limbus, and the inferior spiral ligament (Supplemental Figure 4). These findings suggest that SGNs are the primarily cell type affected by proteotoxic stress in the cochlea in response to noise exposure.

We next examined whether prophylactic administration of TRC051384 could reduce GFP accumulation. In line with its role as a heat shock response activator, TRC051384 significantly attenuated noise-induced UPS dysfunction, as reflected by diminished GFP signal intensity in the spiral ganglion (Figure 4D). However, the increased GFP signal observed in the axons and stria vascularis may indicate that intense noise exposure combined with TRC051384 induces off-target cellular stress within the cochlea. Such stress could increase tissue autofluorescence or promote nonspecific accumulation of GFP. To confirm that the GFP signal originated from the somata of SGNs, we performed immunostaining on the same sections using NF200 antibodies and observed strong colocalization (Figure 4E). Quantitative analysis revealed that GFP signal intensity was significantly reduced in mice treated with TRC051384 before noise exposure (0.12 ± 0.048) relative to vehicle-treated controls (0.37 ± 0.10) (Figure 4F). Further validation of the results was achieved through Western blot analysis, which showed that TRC051384 reduces the accumulation of GFP in cochlear extracts after noise exposure. Treated mice exhibited a reduced signal intensity (1.82 ± 0.002) compared with untreated controls (2.17 ± 0.03) (Figure 4, G and H). These findings together demonstrate that the administration of TRC051384 as a prophylaxis activates the heat shock pathway, preserving the function of the UPS in the cochlea following noise exposure.

### Administration of TRC051384 prior to noise exposure protects against hearing loss.

Hsf1 expression was previously found to be necessary for threshold recovery following noise exposure (35). However, the therapeutic potential of activating Hsf1 with small molecules to prevent hearing loss has received little attention. To explore this possibility, we investigated whether TRC051384-mediated activation of the Hsf1 pathway prior to noise exposure could confer protection in 16-week-old CBA/CaJ mice. TRC051384 (60 mg/kg) or vehicle alone was injected i.p. 2 hours before exposure to 100 dB SPL noise for 2 hours, and ABRs were measured at 1, 3, and 14 DAN. Acute threshold shifts were observed in the TRC051384 and vehicle-treated mice at 1 DAN (Figure 5A). In the vehicle-treated cohort at 3 DAN, thresholds remained significantly elevated at frequencies of 24 kHz and greater, and remained slightly elevated at 14 DAN. However, in the TRC051384-treated cohort, threshold levels fully recovered by 3 DAN at all frequencies except 32 kHz, which then fully recovered by14 DAN. ABR Wave I amplitudes in the vehicle cohort generally failed to recover at intensities > 80 dB SPL at 16 and 32 kHz by 14 DAN (Figure 5, B–D). In comparison, in the TRC051384-treated cohort, ABR Wave I amplitudes fully recovered by 3 DAN. These findings were corroborated by an independent replication cohort of 16-week-old CBA/CaJ mice, which revealed that TRC051384 treatment resulted in markedly greater Wave I amplitude recovery compared with vehicle controls by 14 DAN at 24 and 32 kHz (Supplemental Figure 5, A and B). To evaluate whether TRC051384 protects cochlear synapses, we compared ribbon synapse density between drug-treated and vehicle control groups by immunostaining cochlear whole mounts at 14 DAN with CtBP2 and GluA2 antibodies (Figure 5E). Consistent with TRC051384’s ability to mitigate noise-induced reductions in Wave I amplitude, the decrease in synapse density observed in vehicle-treated CBA/CaJ mice at 14 DAN was absent in TRC051384-treated mice (Figure 5F). In summary, prophylactic treatment with TRC051384 preserves cochlear synapse density and auditory nerve function, supporting its potential as a protective intervention against NIHL.

## Discussion

These findings offer insight into previously unrecognized mechanisms of cochlear proteostasis underlying NIHL. We first confirmed that a widely used noise exposure protocol caused nearly pure TTS in 16-week-old CBA/CaJ mice (22). The difference in noise susceptibility between 8- and 16-week-old CBA/CaJ mice is consistent with several previous studies showing that vulnerability to PTS falls across this age window in CBA-derived strains (36–39), and 6-week-old mice given a similar 100 dB SPL exposure sustain permanent rather than temporary shifts (40). While the basis is unresolved, neither middle ear transmission nor baseline sensitivity changes noticeably across these ages (36), changes in endocrine maturation or an age-dependent shift in the site of noise injury are potential explanations (37). Thus 16-week-old mice were used in all subsequent experiments in our study. We performed bulk discovery-based RNA-seq of cochlear extracts to determine the temporal dynamics of noise-induced gene expression. We detected a pronounced transcriptional response 4 hours after exposure, which represented the peak of gene expression. DAVID enrichment analysis identified the ER as the intracellular structure where the most significantly upregulated gene enrichment occurred (159.7-fold enrichment; *P* = 8.95 × 10^–^¹¹; FDR = 1.09 × 10^–9^), indicating that ER stress is the most prominently engaged cellular response based on gene expression changes. This enrichment was driven by elevated expression of *Hsp90b1* (Endoplasmin), *Hspa5* (BiP), *Dnajb11* (ERdj3), *Dnajc13* (RME-8), and *Sdf2l1* (BiP cochaperone). Among these, *Hsp90b1*, *Hspa5*, *Dnajb11*, and *Sdf2l1* encode ER-resident chaperones and cochaperones involved in protein folding and the UPR, while *Dnajc13* is implicated in endosomal trafficking and membrane dynamics. Spatial transcriptomic profiling revealed that the proteotoxic stress response was particularly pronounced in SGNs and their neighboring supportive cells. These cells exhibited elevated expression of genes encoding proteasome subunits, suggesting enhanced proteostasis activity.

To evaluate the potential protective effects of prophylactic HSR activation against noise-induced cochlear injury, we used TRC051384, a previously characterized Hsf1 activator, which was administered prior to noise exposure. Using a fluorescent UPS reporter mouse model, we mapped the prominent localization of proteostatic stress and found it to be concentrated in the spiral ganglion. Notably, TRC051384 pretreatment reduced accumulation of the GFP-based UPS reporter, suggesting improved proteasomal function and overall protein QC under noise-induced stress conditions. Finally, this pharmacological intervention produced functional and structural benefits. ABR Wave I amplitudes showed enhanced recovery, and synaptic integrity was better preserved. Taken together, these results underscore the potential of heat shock pathway modulation as a prophylactic strategy against NIHL.

Our findings represent a significant advance in understanding a key mechanism underlying NIHL, while also building on our and others’ prior work in the field. Pioneering work from over 20 years ago first identified a link between noise trauma and the proteostasis network through increased gene expression of the E3 ubiquitin ligase *Ube3b* (41). Furthermore, UBE3B is selectively expressed in SGNs in the human cochlea, supporting our finding that SGNs are a key cell type experiencing proteotoxic stress following noise exposure (42). Further evidence supporting the involvement of noise-induced impaired proteostasis, specifically in SGNs, comes from single-cell transcriptomic analyses. These analyses identified *DDIT3* (i.e., *CHOP*), a key transcription factor that regulates ER stress and apoptosis, as being significantly elevated in this cell population (43). Our discovery that activating the HSR with TRC051384 mitigates noise-induced damage aligns with a substantial body of work implicating Hsp in cochlear protection. For example, acutely elevating body temperature in CBA/CaJ mice reduces noise-induced threshold shifts via HSR activation (44), and mice lacking Hsf1 show impaired threshold recovery after noise overstimulation (35), establishing that this protein chaperone pathway is not merely correlated with but also required for normal recovery. At the effector level, induction of HSP70 expression can protect HCs from ototoxic injury (45), and supporting cells can secrete HSP70 to protect neighboring HCs in a non-cell-autonomous manner (46). Consistent with a protective role in humans, polymorphisms in *HSPA1A* and *HSPA1L* have been associated with susceptibility to NIHL in noise-exposed workers (47). The most extensively characterized pharmacological approach, geranylgeranylacetone, induces HSPs through a broad and incompletely defined mechanism (48); TRC051384 offers a potentially more selective route to HSF1 activation, and our results suggest that is sufficient to confer protection when delivered prophylactically. Our findings also support and extend previous work demonstrating the importance of ER stress and the activation of the UPR in response to noise exposure, particularly through the ER-resident protein Tmtc4 (49). Furthermore, prior studies have shown that pharmacological activation of eIF2B, which suppresses the UPR, can mitigate NIHL (49, 50). More recently, mutations in the transcription factor ATF6, which regulates a gene expression program critical for maintaining ER function and initiating the UPR, have been linked to a deafness-blindness syndrome (51, 52).

As with any study, our work has limitations that warrant consideration. First, the initial RNA-seq analysis revealed strong, specific upregulation of genes encoding components of the ER chaperone complex. However, this bulk approach is biased toward changes in gene expression in the most abundant cochlear cell types, which limits resolution. Another limitation of the present study is that the RNAscope analysis was performed following a 105 dB SPL noise exposure that produces PTS, whereas the other experiments used a 100 dB SPL exposure associated with TTS. The higher noise intensity was necessary to generate a sufficiently robust signal for spatial analysis; however, this difference in exposure severity should be considered when comparing the RNAscope findings with the remainder of the study. A key limitation of our spatial transcriptomic analysis was the absence of probes targeting many ER chaperone genes. This prevented us from directly assessing their expression at the level of individual cell types. Despite considerable effort, we were unable to obtain sufficient transcriptomic signal from IHCs, OHCs, and supporting cells within the organ of Corti, likely due to the platform’s limited sensitivity (53). However, in our previous studies, we showed that both Hsp90b1 and Psmc5 have elevated levels in HCs based on immunofluorescence microscopy (26). It is also important to note that, while our previous findings show a noise intensity–dependent increase in the abundance of many proteins (26), suggestive of overproduction or stress-induced misfolding, additional cellular stressors likely contribute to this response. In particular, oxidative stress and disrupted calcium homeostasis are well-known inducers of ER chaperone expression and likely contribute in shaping the complex, multifactorial response to auditory trauma (54). While we demonstrated that TRC051384 activates expression of the Hsf1 target gene, *Hspa1a*, in both heterologous cells and cochlear organotypic cultures, we acknowledge that we did not directly confirm activation of the HSR using qPCR in vivo within the intact mouse cochlea. Although the UPS fluorescent reporter mouse offered a sensitive tool to identify cells experiencing proteotoxic stress and to assess the mitigating effects of TRC051384, it is important to acknowledge that this represents an artificial reporter system. Furthermore, the UPS-reporter mice were maintained on a C57BL/6 background, whereas the principal noise-exposure and TRC051384 studies were performed in male CBA/CaJ mice; strain-dependent differences in auditory susceptibility and proteostasis may therefore influence the magnitude of the observed responses.

Looking ahead, several key avenues should be pursued to build on our findings. First, although our results revealed that SGNs are a key cell type undergoing proteotoxic stress following acoustic trauma, further studies are needed to confirm and characterize this response across additional cochlear cell types. Second, additional mechanistic studies are needed to understand how the heat shock response intersects with other cellular stress pathways such as the UPR and how it integrates stress responses, oxidative stress, calcium dysregulation, and mitochondrial dysfunction. Third, although TRC051384 showed promising protective effects in mice, future studies will be needed to evaluate their pharmacokinetics, cochlear bioavailability, and potential off-target effects. It is also important to note that the precise proximal molecular mechanism by which TRC051384 activates HSF1 in the cochlea is not well established. Testing this compound or improved Hsf1 modulators in other mouse strains and in larger animal models (e.g., guinea pigs or nonhuman primates) is a critical next step for advancing its translational potential. Additionally, studies exploring optimal timing, dosage, and delivery routes are also necessary to maximize therapeutic efficacy and minimize risk. Finally, future studies should investigate whether activating the HSR after noise exposure can confer therapeutic benefits. These efforts will collectively help to clarify the feasibility of targeting cochlear proteostasis as a preventive or therapeutic strategy for NIHL in humans.

## Methods

### Sex as a biological variable.

Mice and rats of mixed sexes were used in our experiments; however, the presented results were not sufficiently powered to determine sex-specific effects. Male mice were used in Figure 1 and Figure 5.

### Mouse procedures.

ABRs and DPOAEs were recorded from young adult mice (approximately P56 or P112). All recordings were performed in a sound-attenuated chamber (Eckel Audiometric Room, Cambridge, MA) using a Tucker-Davis Technologies (TDT) System III workstation running BioSigRP software. ABR stimuli were preamplified using a 4-channel MEDUSA preamplifier and processed in real-time by the TDT System III. Prior to electrode placement, animals were anesthetized via intraperitoneal injection of ketamine (100 mg/kg) and xylazine (3 mg/kg). Supplemental injections (25% of the initial dose) were administered as needed to maintain anesthesia. Core body temperature was maintained at 37°C–38°C using an electric heating pad (Homeothermic Blanket System, Harvard Apparatus). Platinum subdermal needle electrodes were placed at the vertex (recording), ipsilateral mastoid (reference), and lower back (ground). ABRs were evoked by 20-millisecond tone pips (8–32 kHz, 4 kHz increments) at sound levels from 10 to 90 dB SPL, with 5 dB steps from 10–40 dB and 10 dB steps from 40–90 dB. Responses were averaged across 500 repetitions. ABR thresholds were defined as the lowest sound level eliciting a detectable Wave I with an amplitude at least 0.18 μV above the noise floor. Wave I amplitude was measured as the voltage difference between the Wave I peak (P1) and the following trough (N1). For DPOAE recordings, an Etymotic ER-10B+ probe microphone system was used. Stimuli consisted of 2 primary tones, f1 and f2, with a frequency ratio of f2/f1 = 1.2. The sound-pressure levels of the primary tones, L1 and L2, were varied from 15 to 75 dB SPL in 5 dB increments, with L1 maintained 5 dB above L2. Measurements were obtained across f2 frequencies from 8 to 32 kHz. The spectral amplitudes of f1, f2, the 2f1–f2 distortion product, and the surrounding noise floor were calculated from responses averaged over 500 presentations.

Noise exposures were delivered to unanesthetized animals placed in small wire cages within a custom-built sound-attenuated chamber designed by Charles Liberman (Mass. Eye and Ear). The chamber was constructed from 3/4-inch plywood sheets, with the key design principle being that no 2 sides were parallel, minimizing acoustic reflections. The front and back panels were identical in shape, except that the rear panel was truncated at a height of 42 inches, creating a slanted top surface relative to the floor. Side panels were cut to fit this asymmetrical geometry. The top panel included a rectangular cutout for mounting an exponential horn and acoustic driver. Acoustic overexposure waveforms were generated using a TDT waveform generator (Alachua, Florida, USA), producing band-limited noise spanning 8–16 kHz for a total duration of 30 minutes or 2 hours. Noise was delivered via an exponential horn connected to a JBL 2446H/J compression driver (Northridge, CA). Mice were exposed to calibrated noise levels ranging between 94 and 105 dB SPL (1.00 Pa Sound Intensity Level [SIL]). SPL stability was monitored in real time using a PicoLog system (Picoscope 2000 series) and a calibrated microphone (PCB Piezotronics, NY).

B6.Cg-Tg(CAG-Ub*G76V/GFP)1Dant/J, (Strain #:008111, RRID:IMSR_JAX:008111) were purchased from The Jackson Laboratory (Jax).

TRC051384 was prepared in a vehicle consisting of 12% dimethyl sulfoxide (DMSO) and 88% vehicle #1 (10% DMSO/30% Kolliphor EL/10% Ethanol/50% PBS), and the solution was filtered to sterilize. The compound was administered to mice via i.p. injection at a dose of 60 mg/kg. Injections were carried out under aseptic conditions using standard i.p. techniques to ensure accuracy and reproducibility.

### Bulk RNA-seq and qPCR.

For Bulk RNA-seq, total RNA was extracted from cochlear tissue using TRIzol reagent (Thermo Fisher Scientific) according to the manufacturer’s protocol and further purified with the RNeasy Plus Mini Kit (Qiagen). Purified RNA was sent to Novogene (Sacramento, CA, USA) for QC, library preparation, sequencing, and quantitation according to their standard gene expression workflow. RNA degradation and contamination were initially assessed on 1% agarose gels. RNA purity was evaluated using a NanoPhotometer spectrophotometer (IMPLEN, CA, USA), while RNA integrity and concentration were determined using the RNA Nano 6000 Assay Kit with the Bioanalyzer 2100 system (Agilent Technologies, CA, USA). Only RNA samples that passed QC were used for library preparation and sequencing.

For library construction, 1 μg of total RNA per sample was used as input. Libraries were prepared with the NEBNext Ultra RNA Library Prep Kit for Illumina (NEB, USA) according to the manufacturer’s instructions, with unique index codes added to each sample. Briefly, mRNA was enriched using poly-T oligo-attached magnetic beads, fragmented under elevated temperature in First Strand Synthesis Reaction Buffer (5X), and reverse-transcribed using random hexamer primers and M-MuLV Reverse Transcriptase (RNase H–). Second-strand cDNA synthesis was performed with DNA Polymerase I and RNase H, followed by end-repair and adaptor ligation with NEBNext adaptors containing a hairpin loop structure. cDNA fragments of 150–200 bp were size-selected using the AMPure XP system (Beckman Coulter, USA). USER Enzyme (NEB, USA) treatment was performed at 37°C for 15 minutes, followed by 95°C for 5 minutes. Libraries were amplified with Phusion High-Fidelity DNA Polymerase, Universal PCR primers, and Index primers. PCR products were purified with AMPure XP, and library quality was verified on the Agilent Bioanalyzer 2100 system. Indexed libraries were clustered on a cBot Cluster Generation System with PE Cluster Kit cBot-HS (Illumina), and paired-end sequencing (2 × 150 bp) was performed on an Illumina NovaSeq 6000 platform.

Raw FASTQ reads were processed with fastp to remove adaptor sequences, poly-N reads, and low-quality reads. Clean data quality metrics (Q20, Q30, and GC content) were assessed. Reference genome and annotation files were obtained from NCBI/UCSC/Ensembl. Genome indexing and alignment were performed with STAR (v2.5) using the Maximal Mappable Prefix (MMP) algorithm, achieving > 95.3% total mapping efficiency and > 75 million mapped reads per sample across 4 biological replicates per group. Gene-level counts were obtained with HTSeq (v0.6.1).

Differential gene expression analysis was conducted using iGEAK (R/Shiny-based pipeline). Low-abundance transcripts (read count < 8 in fewer than 3 samples) were filtered out. Data were normalized using edgeR’s TMM (Trimmed Mean of M-values) method. Statistical testing for differential expression was performed with edgeR, and *P* values were adjusted for multiple testing with the Benjamini–Hochberg method to control the FDR. Genes with adjusted *P* < 0.05 and fold-change > 1.5 were considered significantly differentially expressed.

qPCR was performed as follows. Total RNA was isolated using TRIzol reagent (Thermo Fisher, 15596026) and purified with the RNeasy Kit (Qiagen, 74104). cDNA was synthesized from isolated RNA using the iScript cDNA Synthesis Kit (Bio-Rad, 1708890). qPCR was carried out on a QuantStudio 3 Real-Time PCR System (Thermo Fisher, A28567) using Power SYBR Green Master Mix (Thermo Fisher, A46109). Each reaction contained 50 ng of cDNA. The thermal cycling conditions were as follows: initial denaturation at 95°C for 10 minutes, followed by 40 cycles of 95°C for 15 seconds and 60°C for 60 seconds. Primers were purchased from IDT and reconstituted to a 10 μM working concentration. The following primer sequences were used (5′–3′): Hsf1 (rat) forward, ATGCCATGGACTCCAACCTG; Hsf1 (rat) reverse, CTCCTGAATGTCCAGCAGGG; Hspa1a (rat) forward, TGATCGCCGGTCTAAACGTG; Hspa1a (rat) reverse, CCCAGGTCGAAGATGAGCAC, GAPDH (rat) forward, GGTGTGAACGGATTTGGC; GAPDH (rat) reverse, ATGTAGTTGAGGTCAATGAAGGG; Hsf1 (human) forward, GAAGCAGCTGGTGCACTACA; Hsf1 (human) reverse, AAGTAGGAGCCCTCTCCCAG; Hspa1a (human) forward, GAGGGCCATGACGAAAGACA; Hspa1a (human) reverse, GCTCGATCTCCTCCTTGCTC; GAPDH (human) forward, CCCACTCCTCCACCTTTGAC; and GAPDH (human) reverse, GAGATTCAGTGTGGTGGGGG. HEK293T cells were purchased from ATCC (catalog CRL-3216) and cultured under standard conditions in DMEM supplemented with 10% fetal bovine serum and penicillin-streptomycin-glutamine. Organotypic cochlear cultures were prepared from P4 rat pups. Cochleae were dissected in ice-cold HBSS, and the basilar membrane containing the organ of Corti, and associated spiral ganglion tissue was isolated and positioned on collagen-coated culture dishes. Explants were maintained in serum-free DMEM/F12 supplemented with N2, B27, and penicillin-streptomycin at 37°C in a humidified atmosphere containing 5% CO_2_. Cultures were allowed to equilibrate overnight before experimental treatment, and the medium was replaced every 2 days. For in vitro experiments, TRC051384 was dissolved in 100% DMSO and applied at a final concentration of 12.5 μM, corresponding to 0.05% DMSO in the culture medium.

### Spatial transcriptomics.

Spatial transcriptomic profiling was conducted using the NanoString GeoMx Digital Spatial Profiler at the Northwestern University Immunotherapy Assessment Core (IAC) following the manufacturer’s guidelines. Mouse cochleae were recovered from sham or treated mice and embedded in OCT. Cochleae were sectioned at a thickness of 6 μm and processed for the GeoMx Mouse Whole Transcriptome Atlas (GeoMx NGS RNA WTA Mm; NanoString/Bruker Spatial Biology) according to the manufacturer’s instructions with an exception of 0.0001% Proteinase K used in slide preparation step. ROI were selected in the “tissue area name”. Cells were collected in this order: (a) NF200^+^ and (b) NF200^–^ cells with a target of at least 50 events per ROI. Segmentation was performed with the following settings: 0 Erode, 2 μm N-Dilate, 100 μm2 Hole Size, and 5 μm2 Particle Size. After construction and QC (Qubit and bioanalyzer) of the GeoMx library, single-end 50 nucleotide sequencing was performed on a Hiseq 4000 System (Illumina) with a total of 100 million reads. After sequencing, the NanoString GeoMx Digital Spatial Profiler (DSP) with DRAGEN pipeline were used to automatically process the Illumina FASTQ sequencing files to GeoMx readable digital counts conversion files for input back into the DSP Data Analysis Suite. Data comparisons represent total cells pooled from 21 ROI for the noise exposed group and 30 ROI from the control group.

### NGS library preparation and sequencing.

QC for the constructed library was performed using the Agilent Bioanalyzer High Sensitivity DNA kit (Agilent Technologies, 5067-4626) and the Qubit DNA HS assay kit for qualitative and quantitative analysis, respectively. A Qubit concentration of 0.156 ng/μL had an average fragment size of 182 bp. The final library was sequenced on Illumina HiSeq4000 sequencer using single end 50 nt mode.

### MS analysis.

Cochlear extracts were prepared as follows. Two cochleae from control 4-month-old C57BL/6NJ mice were solubilized in 100 μL of 2% acetonitrile (ACN) buffer containing 0.05% trifluoroacetic acid (TFA) using a Precellys homogenizer and incubated on ice for 5 minutes. Samples were centrifuged at 12,000*g* for 15 minutes, and the supernatant was transferred to a fresh tube. Four volumes of ACN were added, mixed thoroughly, and incubated on ice for 5 minutes. Following a second centrifugation at 12,000*g* for 15 minutes, the supernatant was collected and dried in a SpeedVac concentrator. The dried pellet was resolubilized in 50 μL of buffer A (94.785% H_2_O, 5% ACN, 0.125% formic acid [FA]), and 15 μL was injected for LC-MS/MS analysis. Samples were loaded via autosampler using a Thermo EASY-nLC 1000 UPLC pump onto a vented Acclaim PepMap 100 trap column (75 μm × 2 cm, Thermo Fisher Scientific) connected to an Acclaim PepMap 100 nanoViper analytical column (75 μm × 500 mm, 3 μm particle size, 100 Å pore size, C18; Thermo Fisher Scientific, 164570) with a stainless-steel emitter tip. The column was interfaced with a Nanospray Flex Ion Source operating at a spray voltage of 2,000 V. Mass spectra were acquired on an Orbitrap Fusion mass spectrometer (Thermo Fisher Scientific). Buffer A consisted of 94.785% H_2_O, 5% ACN, and 0.125% FA, while buffer B consisted of 99.875% ACN with 0.125% FA. Peptides were separated over a 60-minutes gradient with the following profile: 2%–8% buffer B (3 minutes), 8%–24% (32 minutes), 24%–36% (10 minutes), 36%–55% (5 minutes), and 55%–95% (5 minutes), followed by 95% (5 minutes).

We analyzed 50 pg of TRC051384 (TRC051384, 867164-40-7) resuspended in Buffer A. MS methods were as follows. Targeted SIM (tSIM) was used for quantification. For MS1 quantitation, the doubly charged precursor ion ([M+2H]²^+^) at *m/z* 233.6261 was used (neutral monoisotopic mass, 465.23 Da). For MS2, quantitation was performed on the same precursor with multiple fragment ions analyzed. MS data were manually inspected using Excalibur software, and MS2 spectra were further processed with Compound Discoverer (Thermo Fisher Scientific). For MS1, precursor ions were isolated using the quadrupole with a 20 *m/z* isolation window, and detection was performed in the Orbitrap at 120,000 resolution (positive ion mode). For MS2, precursor isolation was carried out with a 1.6 *m/z* isolation window, followed by HCD fragmentation at 30% normalized collision energy. Detection was performed in the Orbitrap at 30,000 resolution with RF Lens set to 30% (positive ion mode).

### Western blots.

Cochleae were rinsed in PBS and homogenized in Syn-PER buffer (Thermo Scientific, 87793) supplemented with 1% Triton X-100 (Sigma-Aldrich, T8787) and 0.5% SDS using a Percellys 24 tissue homogenizer (Bertin, P000669-PR240-A). Homogenization was performed at room temperature with three 30-second pulses at 6,800 rpm. Lysates were incubated on ice for 10 minutes. Samples were then incubated on ice for an additional 30 minutes, followed by centrifugation at 10,000*g* for 5 minutes at 4°C. The resulting supernatants were collected, and protein concentrations were determined using the Pierce BCA Protein Assay Kit (Thermo Fisher Scientific, 23225). For immunoblotting, 25 μg of cochlear lysate was mixed with 6× loading buffer, boiled at 100°C for 5 minutes, and immediately loaded onto a 10% Tris gel (Bio-Rad). Proteins were electrophoretically separated and transferred to nitrocellulose membranes using the Bio-Rad Trans-Blot Turbo transfer system (1.3 A, 25 V, 10 minutes). Membranes were blocked with 1× Odyssey blocking buffer (LI-COR, 927-70001) for 1 hour at room temperature, then incubated overnight at 4°C with primary antibodies GFP (1:500, Sigma, SAB4301138, RRID: AB_2750576) and GAPDH (Cell Signaling Technology, 2118S, RRID: AB_561053). After 3 washes in TBST, membranes were incubated with secondary antibodies for 2 hours at room temperature. Blots were imaged using the Odyssey DLx Imaging System (LI-COR).

### Fluorescence microscopy.

Mice were sacrificed, and temporal bones/cochleae were isolated within 3 minutes of euthanasia. Samples were rinsed in ice-cold PBS supplemented with Halt protease inhibitor cocktail until the buffer remained clear of blood after vortexing. The oval and round windows were punctured, and a small hole was made near the apex of the cochlea to gently perfuse the scalae with PKS solution (4% paraformaldehyde, 126 mM NaCl, 2.5 mM KCl, 25 mM NaHCO_3_, 1.2 mM NaH_2_PO_4_, 1.2 mM MgCl_2_, 2.5 mM CaCl_2_, 11 mM sucrose). Samples were then immersed in PKS and fixed for 1 hour at 4°C. Cochleae were decalcified in Immunocal (Decal Chemical Corp., Congers, NY) for ~2 hours at 4°C, or until translucent. The decalcified bone, lateral wall, Reissner’s membrane, and tectorial membrane were carefully dissected away from the organ of Corti. For whole-mount preparations, cochleae were divided into 4 pieces, cryoprotected in 30% sucrose for 30 minutes, and frozen on dry ice. Frozen tissues were thawed and rinsed 3 times in PBS for 10 minutes each at room temperature with agitation.

Cochlear pieces were blocked overnight at 4°C in blocking buffer (20% normal horse serum, 1% Triton X-100 in PBS). Primary antibodies were diluted in blocking buffer as follows: Recombinant Neurofilament H (NF200) (1:1,000, Synaptic Systems, 171106, RRID: AB_2721078), CtBP2 (1:200, Thermo, 612044, RRID: AB_399431), GluA2 (1:100, Chemicon, MAB397, RRID: AB_2113875), and Myo7a (1:500, Thermo, PA1-936, RRID: AB_2235704). Samples were incubated with primary antibodies at 37°C overnight, rinsed 3 times in PBS (15 minutes each), and then incubated with secondary antibodies for 3 hours at room temperature: Goat Anti-Mouse IgG2a Alexa Fluor 488 (1:500, Abcam A21131), Goat Anti-Mouse IgG1 Alexa Fluor 568 (1:500, Abcam A21124), Goat Anti-Rabbit H&L Alexa Fluor 647 (1:250, Abcam A27040), Donkey Anti-Sheep H&L Alexa Fluor 647 (1:250, Abcam ab150179), and Donkey Anti-Sheep H&L Alexa Fluor 405 (1:250, Abcam ab175676). After secondary incubation, samples were washed 3 times in PBS (15 minutes each) and counterstained with DAPI solution (Abcam ab228549) for 5 minutes. Tissues were mounted using ProLong Gold Antifade Mountant (Thermo Scientific, P10144). Images were captured on a Leica DMI4000 confocal laser scanning microscope under identical acquisition settings.

### RNAscope.

Mouse cochleae were harvested and dissected as described above. Cochleae were fixed for 1 hour, then decalcified overnight in Immunocal. Samples were subsequently cryoprotected overnight in 30% sucrose buffer. Once saturated, cochleae were embedded in OCT and frozen at –80°C. Tissue was sectioned at 12 μm and mounted onto frosted glass slides, which were stored at –80°C until use.

Slides were washed 3 times in PBS at room temperature, followed by sequential dehydration in 30%, 50%, 70%, and 100% ethanol. Slides were then incubated with 3% hydrogen peroxide at room temperature for 10 minutes, treated with RNAscope Protease III for 15 minutes at 40°C, and rinsed 3 times in DEPC-treated water. RNAscope probes were prewarmed at 40°C in a water bath for 10 minutes to dissolve any precipitate, then cooled to room temperature. Target probes for C1, C2, and C3 were mixed in a 50:1:1 ratio, with a final volume of 50 μL per sample. Slides were incubated with the probe mixture for 2 hours at 40°C in the HybEZ Oven. Each channel was then amplified and hybridized to the appropriate fluorophore following the manufacturer’s instructions. Nuclei were counterstained with DAPI. Images were acquired using a confocal laser scanning microscope (Leica DMI4000) under identical acquisition settings. The mouse probes used were as follows: Hspa5 (ACD Bio-Techne, 438831-C), Hsp90b1 ACD Bio-Techne, 556051), Sdf2l1 (ACD Bio-Techne, 562401), Tubb3-C2 (ACD Bio-Techne, 423391-C2), Myo7a (ACD Bio-Techne, 462771), and GFP-C2 - Synthetic construct Cox8ND6gfp fusion protein gene complete cds custom sequence 709/714 (99%).

### Statistics.

Statistical analyses were performed using Excel, GraphPad prism, and SAS 9.4. Data are presented as mean ± SD. For measures obtained across multiple stimulus intensities or frequencies (e.g., ABR thresholds, DPOAE thresholds), comparisons were performed using 1-way ANOVA. Post hoc pairwise comparisons between genotypes were corrected for multiple testing using Tukey’s method. Statistical significance was defined as an adjusted *P* < 0.05.

For the ABR analysis, we use linear mixed effects regression models (LMMs) to account for the correlation between repeated measurements within mouse over time and at different frequencies and intensities. We fit separate models for Noise + Vehicle and Noise + TRC051384. To model changes in threshold by frequency and DAN, we estimated LMMs with threshold as the outcome; fixed effects for frequency and DAN, and their interaction; and a random intercept for mouse to account for repeated measures over time. The resulting β coefficients informed our understanding of how treatment altered hearing recovery at different frequencies over time. For changes in Wave I Amplitude by intensity and DAN, we similarly estimated LMMs with amplitude as the outcome; fixed effects for intensity and DAN, and their interaction; and a random intercept for mouse. We fit these models separately for each frequency (16, 24 and 32 kHz). Statistical comparisons in Figure 1, A and C; Figure 5, A–D; and Supplemental Figure 5, A and B, are all model based.

### Study approval.

All animal experiments were conducted according to Northwestern University IACUC (approved protocol nos. IS00011904, IS00001182, and IS00022569). For euthanasia, pups were immediately decapitated, and adult animals were euthanized by isoflurane overdose and decapitation.

### Data availability.

The RNA-seq datasets supporting the conclusions of this study have been deposited in the NCBI Gene Expression Omnibus (GEO). Bulk RNA-seq data are available under accession number GSE312476, and spatial transcriptomic data are available under accession number GSE312646. Values for all data points in graphs are reported in the Supporting Data Values file.

## Author contributions

JY, MA Ramirez, MA Rutherford, MAC, and JNS designed experiments. JY and MA Ramirez performed ABR and DPOAE experiments. JY, SE, and MA Ramirez performed bulk RNA-seq experiments. MA Ramirez performed spatial RNA-seq experiments. SE performed MS analysis. MA Ramirez, SY, CJL, and YJ performed the in vitro experiments. JY and MA Ramirez performed the IF. MA Ramirez performed the Western blots. JY, MA Ramirez, YZW, SE, MS, LJW, and JNS analyzed the data. MA Ramirez, JY, and JNS wrote the manuscript, and all authors reviewed and edited the manuscript. JY and MA Ramirez are co–first authors; however, JY is the leading first author since he discovered the beneficial effects of TRC051384.

## Conflict of interest

The authors have declared that no conflict of interest exists.

## Funding support

- W81XWH-22-1-0773 and W81XWH-19-1-0627.
- NIDCD grant R01DC014712 (MA Rutherford).

## Supplementary Material

Supplemental data

Unedited blot and gel images

Supplemental table 1

Supplemental table 2

Supplemental table 3

Supplemental table 4

Supporting data values

## Figures and Tables

**Figure 1:**
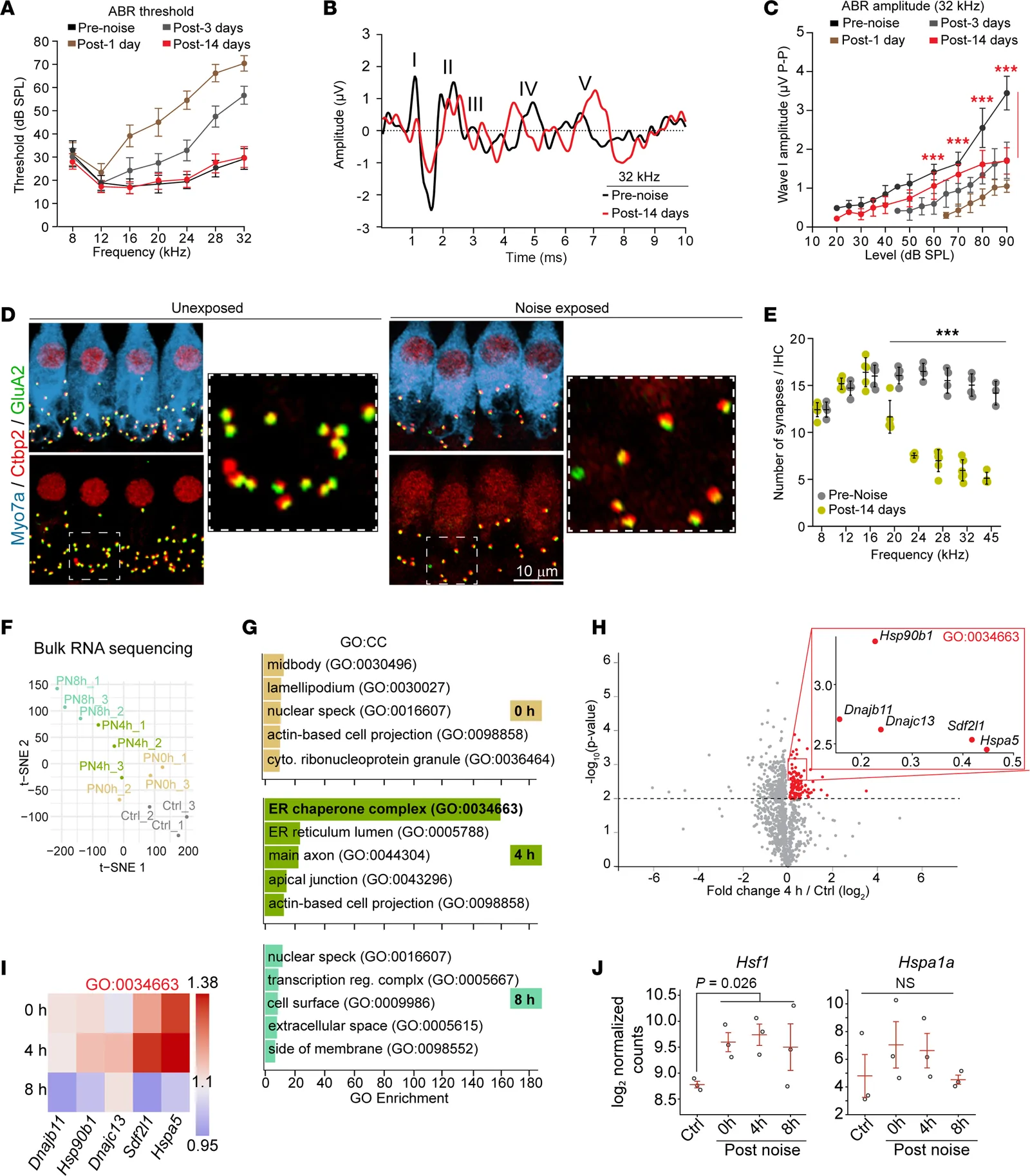
Two-hour exposure to 100 dB SPL noise produces temporary threshold shift, persistent cochlear synaptopathy, and induction of ER chaperone genes in 16-week-old male CBA/CaJ mice. (**A**) ABR thresholds recovered to baseline by 14 days after noise (DAN). (**B**) Representative 32 kHz ABR traces at 90 dB SPL before noise and 14 DAN. (**C**) Wave I amplitudes at 32 kHz remained significantly reduced at 14 DAN across 60–90 dB SPL; differences were estimated by linear mixed modeling. (**D**) Representative 32 kHz cochlear regions immunostained for CtBP2 (red) and GluA2 (green) from unexposed and noise-exposed mice at 14 DAN. Scale bar = 10 μm. (**E**) Quantification of overlapping CtBP2/GluA2 puncta revealed marked synapse loss from 20–45 kHz at 14 DAN (7.45 ± 2.53 synapses/IHC) versus unexposed controls (15.46 ± 0.89). (**F**) t-SNE of bulk cochlear RNA-seq profiles collected immediately, 4 h, or 8 h after noise and from unexposed controls showed clustering by exposure/recovery time. (**G**) Gene Ontology (GO) cellular component (CC) enrichment analysis identified endoplasmic reticulum chaperone complex (GO:0034663) as the most enriched term at 4 h. (**H**) Volcano plot depicting noise-induced changes in cochlear gene expression in the 4-hour postexposure group relative to unexposed controls. Significantly increased genes (*P* < 0.01) are red, with ER chaperone-complex genes highlighted. (**I**) *Hsp90b1*, *Dnajb11*, *Dnajc13*, *Sdf2l1*, and *Hspa5* had elevated expression immediately following noise exposure and peaked at 4 hours postexposure. (**J**) *Hsf1* and *Hspa1a* expression in cochlea. A priori contrasts comparing postnoise time points with controls yielded *P* = 0.026 and *P* = 0.43, respectively. Quantitative data are mean ± SD unless otherwise indicated. **A**–**C**, *N* = 6; **D** and **E**, *N* = 4 control/6 exposed; **F**–**I**, *N* = 4/group; **J**, *N* = 3/group.

**Figure 2:**
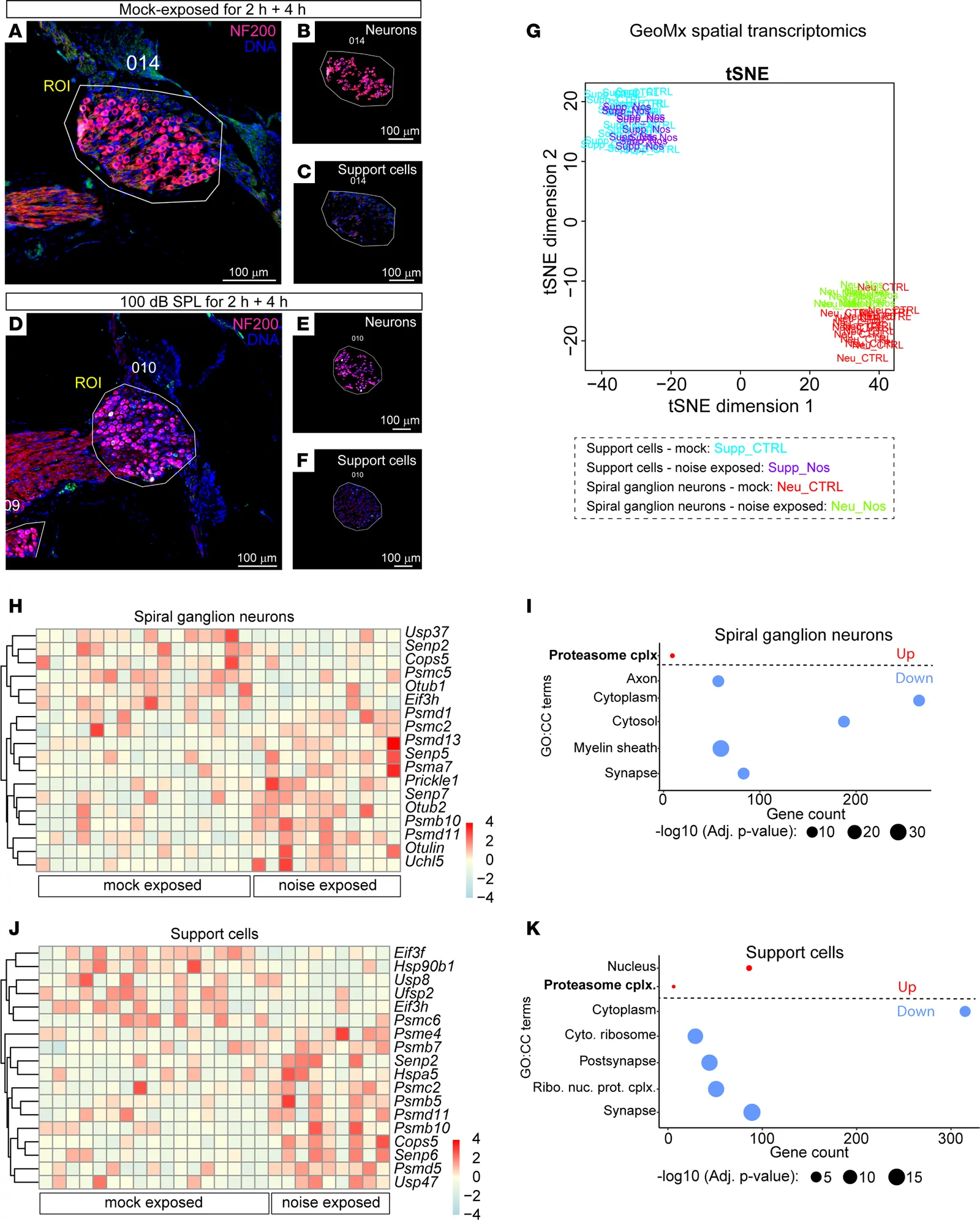
GeoMx spatial transcriptomics reveal that noise exposure selectively upregulates genes encoding multiple proteasome subunits in spiral ganglion neurons and associated support cells. (**A**–**F**) Representative GeoMx immunofluorescent cochlear images from a mock control mouse and an experimental mouse exposed to 100 dB SPL for 2 hours and harvested 4 hours later. NF200^+^ neuronal somata are in magenta; nuclei (Hoechst) are blue. Regions of interest (ROI; white outline) encompassing the spiral ganglion were segmented into a neuronal mask (**B**, NF200^+^) and a support cell mask (**C**, NF200^+^) for probe collection. (**G**) t-SNE plot of RNA-sequencing profiles from individual spiral ganglion neuron ROI, showing that biological replicates cluster primarily by cell type and, to a lesser extent, by noise exposure condition. Supp_CTRL (blue) = supporting cells from mice not exposed to noise, Supp_Nos (purple) = supporting cells from mice exposed to noise, Neu_CTRL (red) = SGNs from mice not exposed to noise, Neu_Nos (green) = SGNs from mice exposed to noise. (**H**) Heatmap showing genes encoding proteostasis-related proteins with significantly altered mRNA levels in spiral ganglion neurons (SGNs). Each row represents a gene, and each column represents a region of interest (ROI). Z-scored expression values are shown, with red indicating upregulation and blue indicating downregulation in response to noise exposure. (**I**) DAVID enrichment analysis of the up-regulated genes in SGNs shows only 1 term that surpasses the significance threshold: the proteasome complex (KW-0647~Proteasome). (**J**) Heatmap showing genes encoding proteostasis-related proteins with significantly altered mRNA levels in support cells. Each row represents a gene, and each column represents a region of interest (ROI). Z-scored expression values are shown, with red indicating upregulation and blue indicating downregulation in response to noise exposure. (**K**) DAVID enrichment analysis of the upregulated genes shows only 2 terms surpassing the significance threshold: the proteasome complex (KW-0647~Proteasome) and nucleus (KW-0539~Nucleus). *N* = 11 (exposed) and 16 (control) ROI for SGNs, *N* = 9 (exposed) and 17 (control) ROI for support cells from *n* = 5 P60–P65 mice of mixed sex per condition.

**Figure 3:**
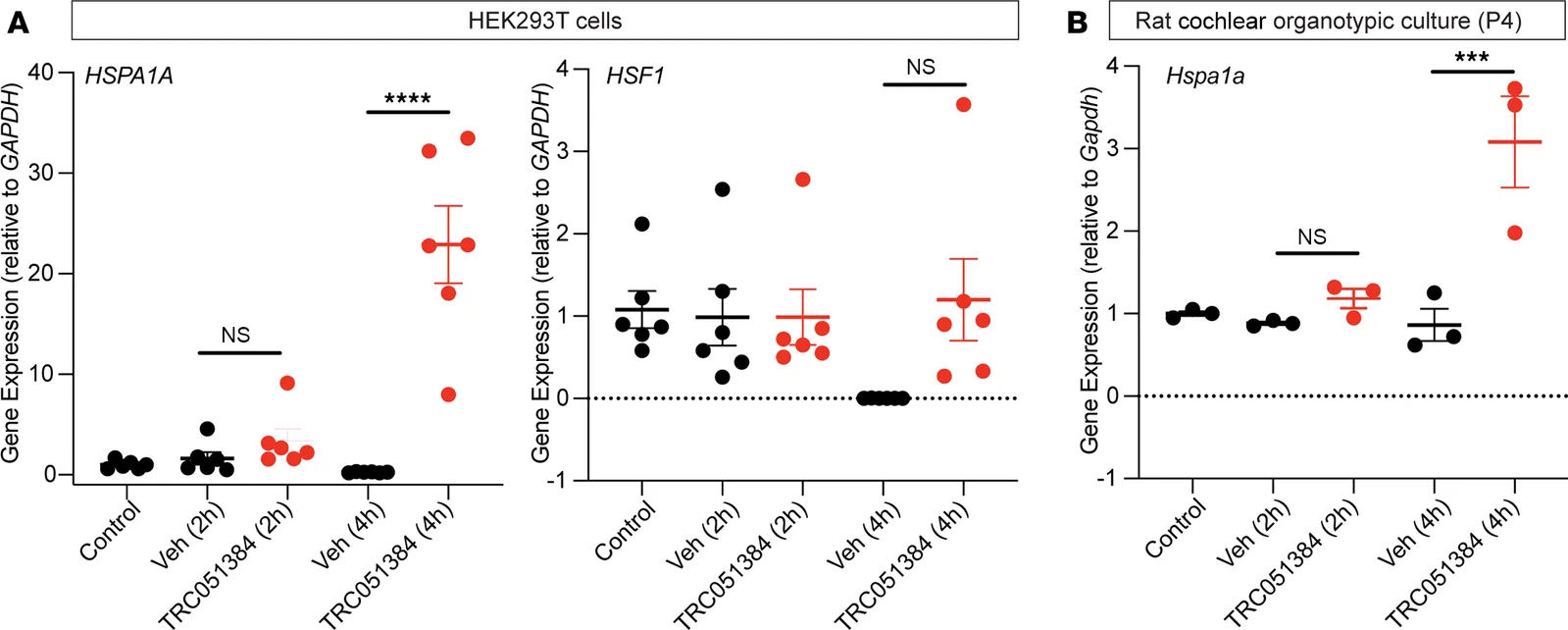
TRC051384 selectively enhances *HSPALA* gene expression without altering *HSF1* expression in HEK293T cells and cochlear organotypic cultures. (**A**) qPCR analysis of RNA from HEK293T cells treated with TRC051384 or with a DMSO vehicle, relative to untreated controls. Cells were untreated (Control) or incubated with DMSO for 2 hours or TRC051384 for 2 or 4 hours prior to RNA collection. *HSPA1A* (heat shock protein family A [Hsp70] member 1A) expression was significantly increased by TRC051384 treatment, with levels rising further at 4 hours relative to DMSO and untreated controls (Control: 1.07 ± 0.45; DMSO: 1.73 ± 1.52; 2 h TRC051384: 3.75 ± 2.88; 4 h TRC051384: 20.10 ± 11.5). *HSF1* mRNA levels remained unchanged across all conditions over the 4-hour period (Control: 1.09 ± 0.55; DMSO: 0.99 ± 0.84; 2 h TRC051384: 1.06 ± 0.91; 4 h TRC051384: 1.18 ± 1.20). *n* = 6 independent biological replicates per condition. (**B**) qPCR analysis of RNA collected from P4 rat cochlear organotypic cultures at DIV3. Cultures were untreated (Control) or incubated with DMSO for 2 hours or TRC051384 for 2 or 4 hours prior to RNA collection. *Hspa1a* gene expression significantly increased by 4 hours relative to DMSO and untreated controls (Control: 1.00 ± 0.06, DMSO 2 hour: 0.89 ± 0.02, DMSO 4 hour: 0.92 ±.32, 2 hour TRC051384: 1.17 ± 0.19, 4 hour TRC051384: 3.08 ± 0.96). *n* = 3 independent cultures per condition. For **A** and **B**, data are represented as mean ± SD, ***P < 0.001 and ****P < 0.0001 by 1-way ANOVA to determine significance with Tukey’s post hoc correction.

**Figure 4:**
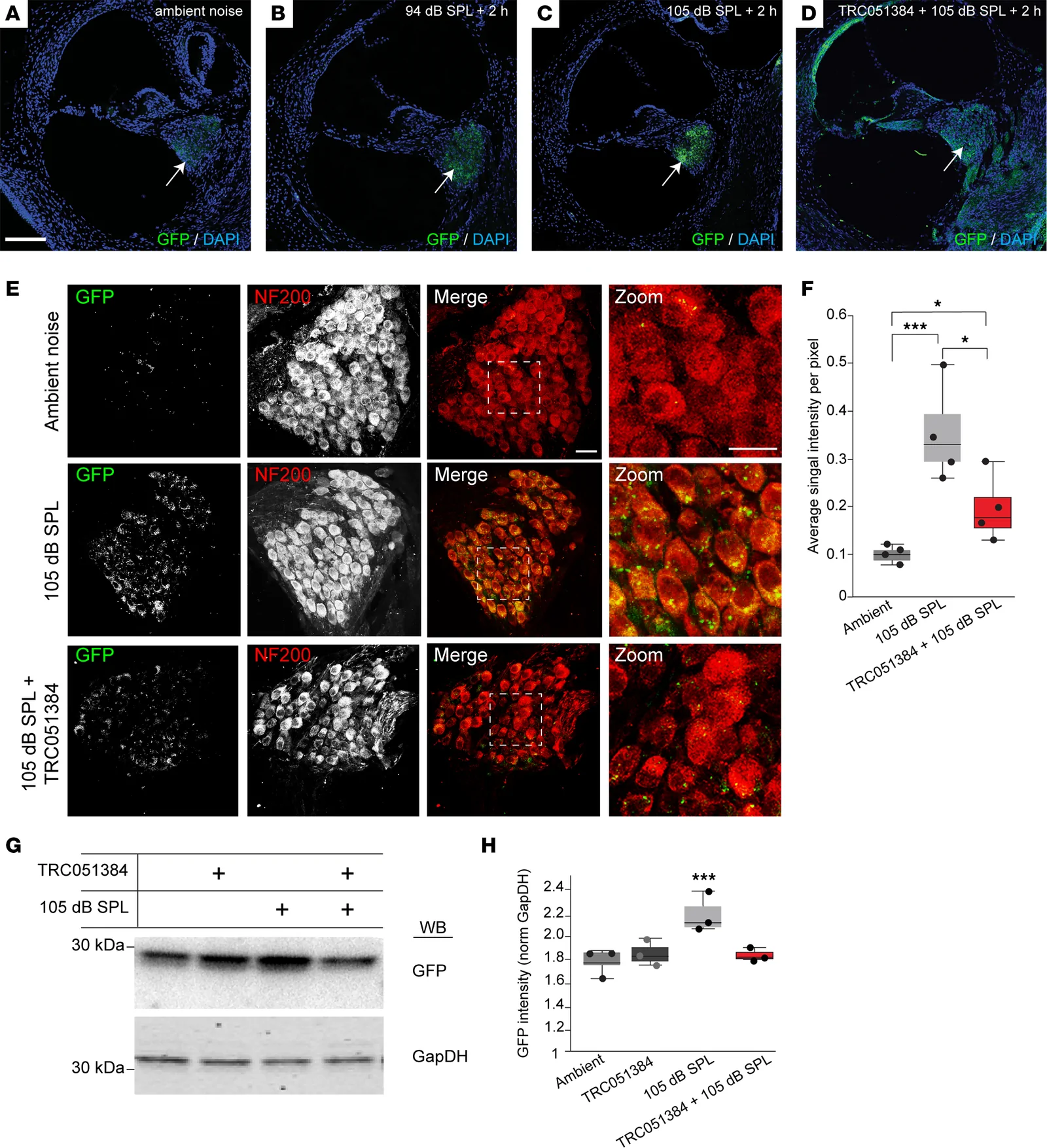
Prophylactic TRC051384 administration attenuates proteasomal impairments in C57BL/6NJ mice after 105 dB SPL noise exposure for 2 hours. (**A**–**C**) Representative fluorescent images from 12 μm cochlear sections near the mid-modiolar plane from P60 Tg(CAG-Ub*G76V/GFP)1Dant mice exposed for 2 hours to 70 dB SPL (ambient), 94 dB SPL, or 105 dB SPL noise, followed by a 2-hour postexposure interval. (**D**) Representative fluorescent images from 12 μm cochlear sections near the mid-modiolar plane from mice pretreated with TRC051384 and subsequently exposed to 105 dB SPL noise for 2 hours, followed by a 2-hour post-exposure interval. Arrows indicate the spiral ganglion region, highlighting GFP-positive signal. (**E**) Representative fluorescent images from 12 μm sections near the mid-modiolar plane, focusing on the spiral ganglion of P60 Tg(CAG-Ub*G76V/GFP)1Dant mice. As expected, noise exposure markedly increased the intensity and abundance of GFP signals within spiral ganglion neurons that were NF200^+^. Prophylactic administration of TRC051384 noticeably reduced both the size and abundance of GFP^+^ clusters. (**F**) Quantification of GFP puncta intensity from **E** shows that noise exposure markedly increased puncta intensity compared with unexposed cochleae (ambient: 0.08 ± 0.024; 105 dB SPL: 0.34 ± 0.11). Prophylactic administration of TRC051384 significantly reduced GFP puncta accumulation following noise exposure, as evidenced by the lower GFP signal in the Noise + TRC051384 group (0.19 ± 0.09). (**G**) Western blot analysis of GFP protein levels in cochlear extracts from mice treated with TRC051384, with or without 105 dB SPL noise exposure for 2 hours. The upper panel shows GFP signal intensity, and the lower panel shows GAPDH as a loading control. (**H**) Quantification of **G** GFP levels normalized to the GAPDH loading control to determine relative GFP signal intensity. TRC051384 treatment alone did not significantly alter GFP abundance compared with uninjected mice (TRC051384: 1.83 ± 0.013; Uninjected: 1.77 ± 0.016). However, TRC051384 significantly reduced GFP abundance following 105 dB SPL noise exposure compared with untreated noise-exposed mice (TRC051384 + Noise: 1.82 ± 0.002; Uninjected + Noise: 2.17 ± 0.031). (**F**) *n* = 4 P60–P65 mice of mixed sex per condition. (**G**) *n* = 3 P60–P65 mice of mixed sex per condition. Data are represented as mean ± SD, *P < 0.05, ***P < 0.001 by 1-way ANOVA determined significance with Tukey’s post hoc correction. Scale bar: 25 μm (**A**–**D**); 15 μm (**E**).

**Figure 5:**
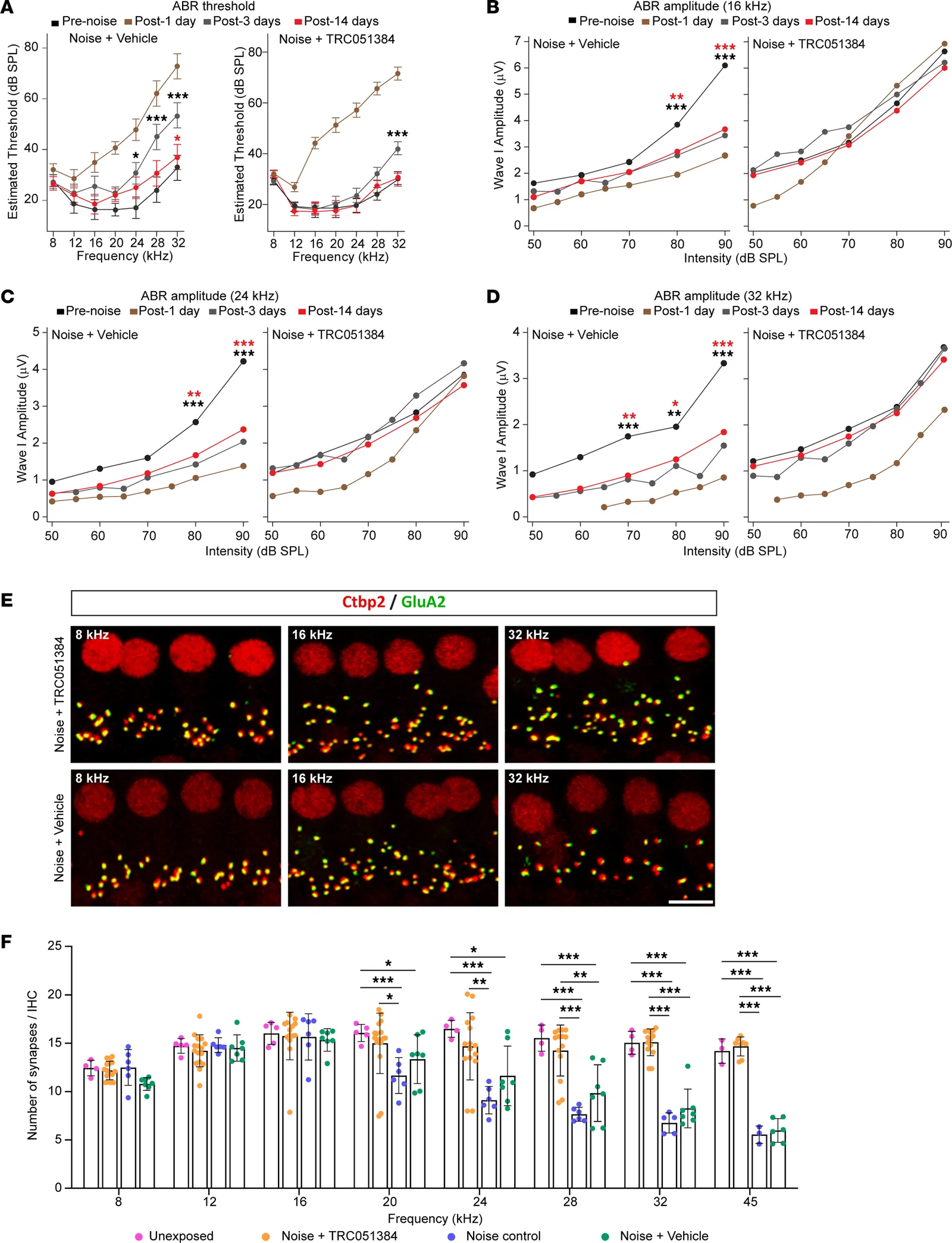
Prophylactic administration of TRC051384 accelerates recovery from noise exposure and prevents cochlear synaptopathy in male 16-week-old CBA/CaJ mice. (**A**) ABR thresholds in vehicle-treated mice remained elevated at 3 days after noise (DAN) and did not fully recover at 32 kHz by 14 DAN. In contrast, TRC051384-treated mice recovered to baseline by 3 DAN at 8 and 16 kHz and by 14 DAN at all frequencies. (**B**–**D**) ABR Wave I amplitudes at 16, 24, and 32 kHz remained persistently reduced in vehicle-treated mice at high stimulus intensities through 14 DAN, whereas TRC051384-treated mice showed recovery by 3 DAN. (**E**) Representative cochlear whole-mount images from the 8, 16, and 32 kHz regions immunostained for CtBP2 (red) and GluA2 (green) in TRC051384- and vehicle-treated mice at 14 DAN. Scale bar, 10 μm. (**F**) Quantification of colocalized CtBP2/GluA2 puncta revealed significantly greater synaptic density in TRC051384-treated mice across most cochlear frequency regions, with the largest differences at higher frequencies. Mean synapses per IHC at 32 kHz were 15.09 ± 1.38 for TRC051384 and 8.26 ± 2.01 for vehicle; at 45 kHz, 14.67 ± 0.98 and 5.99 ± 1.23, respectively. (**A**–**D**) *N* = 17 TRC051384-treated and 7 vehicle-treated mice. (**E** and **F**) *N* = 14–17 TRC051384-treated and 6–7 vehicle-treated mice. (**A**–**D**) Adjusted *P* value * = < 0.05, ** = < 0.01, *** = < 0.001; (**F**) Data are represented as mean ± SD. *P < 0.05, **P < 0.01, ***P < 0.001 by 1-way ANOVA with Tukey’s post hoc correction.

## Version 1 08/25/2026

In-Press Preview

## Version 2 10/08/2026

Electronic publication
