# Supplementary material for: Heat shock pathway activation by TRC051384 protects spiral ganglion neurons from noise-induced hearing loss
Source: JCI Insight. 2026 Aug 25;11(19):e203285. doi: 10.1172/jci.insight.203285 (PMC13726418; doi:10.1172/jci.insight.203285)
Supplement: Supplementary file 2 [file jciinsight-11-203285-s106.pdf]

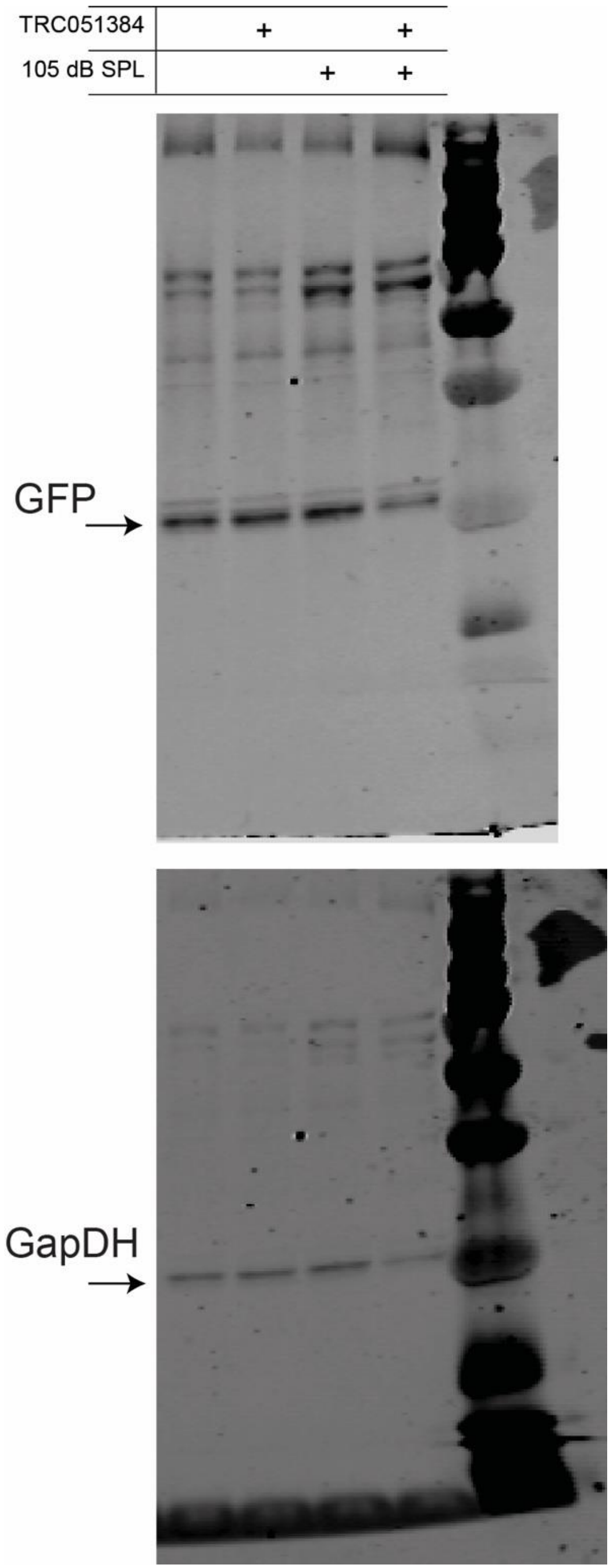
TRC051384 + +
105 dB SPL + +
GFP
GapDH
